# Validity of Simpson-Angus Scale (SAS) in a naturalistic schizophrenia population

**DOI:** 10.1186/1471-2377-5-5

**Published:** 2005-03-17

**Authors:** Sven Janno, Matti M Holi, Katinka Tuisku, Kristian Wahlbeck

**Affiliations:** 1 Department of Psychiatry, University of Tartu, Raja 31, 50417, Tartu, Estonia; 2 Department of Psychiatry, Helsinki University, Helsinki, Finland; 3 Finnish Institute of Occupational Health, Helsinki, Finland; 4 STAKES National Research and Development Centre for Welfare and Health, Helsinki, Finland; 5 Vaasa Central Hospital, Vaasa, Finland

## Abstract

**Background:**

Simpson-Angus Scale (SAS) is an established instrument for neuroleptic-induced parkinsonism (NIP), but its statistical properties have been studied insufficiently. Some shortcomings concerning its content have been suggested as well. According to a recent report, the widely used SAS mean score cut-off value 0.3 of for NIP detection may be too low. Our aim was to evaluate SAS against DSM-IV diagnostic criteria for NIP and objective motor assessment (actometry).

**Methods:**

Ninety-nine chronic institutionalised schizophrenia patients were evaluated during the same interview by standardised actometric recording and SAS. The diagnosis of NIP was based on DSM-IV criteria. Internal consistency measured by Cronbach's α, convergence to actometry and the capacity for NIP case detection were assessed.

**Results:**

Cronbach's α for the scale was 0.79. SAS discriminated between DSM-IV NIP and non-NIP patients. The actometric findings did not correlate with SAS. ROC-analysis yielded a good case detection power for SAS mean score. The optimal threshold value of SAS mean score was between 0.65 and 0.95, i.e. clearly higher than previously suggested threshold value.

**Conclusion:**

We conclude that SAS seems a reliable and valid instrument. The previously commonly used cut-off mean score of 0.3 has been too low resulting in low specificity, and we suggest a new cut-off value of 0.65, whereby specificity could be doubled without loosing sensitivity.

## Background

Reported prevalences for neuroleptic-induced parkinsonism (NIP) in schizophrenia patients are usually in the range 19% to 36% [1-5]. As NIP can severely impair activities of daily life, and it can be treated or at least alleviated, its diagnosis and assessment are an important focus in clinical practice. A reliable diagnosis of NIP is a demanding task [6]. NIP may be missed due to overlap with negative and depressive symptoms in treated schizophrenia patients [7]. Diagnostic and Statistical Manual, fourth edition, (DSM-IV) [8] criteria for NIP consist of parkinsonian tremor, muscular rigidity or akinesia, developing within a few weeks of starting or raising the dose of a neuroleptic medication (or after reducing a medication used to treat extrapyramidal symptoms). Like other motor adverse effects of antipsychotic drugs, the NIP is usually assessed by clinical observation or by rating scales, which are based on clinician's judgement. Movement disorders such as NIP, however, can be measured objectively by recording motor activity [9-12].

Simpson-Angus Scale (SAS) is a 10-item rating scale that has been used widely for assessment of NIP in both clinical practice and research settings [13]. It consists of one item measuring gait (hypokinesia), six items measuring rigidity and three items measuring glabella tap, tremor and salivation, respectively. It is an established rating scale, but some shortcomings have been suggested: the rigidity items may be given too much emphasis, the statistical properties have been studied insufficiently, and the instructions as well as the definitions are somewhat unclear [14]. Several items of the scale have failed to show appropriate interrater reliability or insufficient variability across elderly patients [15], and a modified version has been used to determine the prevalence of spontaneous parkinsonism and the incidence of NIP in this population [16].

According to our recent study [17] there was a discrepancy between SAS and DSM-IV based NIP prevalence estimates. We suggested that the commonly used cut-off point of 0.3 mean SAS score was too low in a naturalistic clinical population [17].

Accelerometric methods have been developed to identify and monitor motor NIP symptoms, such as tremor [18,19] and hypokinesia [20]. A standardized actometric method has been developed for the assessment of neuroleptic-induced akathisia (NIA) [21]. This method discriminated pure NIA patients from healthy controls and from themselves in remission phase with no overlap [21]. In the current clinical population (including patients with NIP and tardive dyskinesia in addition to NIA), however, the method evidenced less diagnostic power [22]. NIP symptoms may have confounded these actometric findings.

The discrepancy between SAS and DSM-IV based NIP prevalence estimates as well as other above mentioned shortcomings suggest that SAS needs an evaluation as a method to assess NIP severity and to find reliably NIP cases.

Our aims were to check the internal consistency of SAS, improve the convergence between DSM IV and SAS based NIP case finding, and to evaluate how well the scale measures objective motor symptoms verified by actometry.

## Methods

We recruited 99 chronic schizophrenic institutionalized adult patients from a state nursing home in central Estonia [17]. Inclusion criteria were DSM-IV diagnosis of schizophrenia or schizoaffective disorder, stable antipsychotic medication (for at least one month), and age of 18–65 years. Diagnosis was made using a semi-structured interview according to DSM-IV criteria for schizophrenia by a psychiatrist (SJ) and medical records. Patients with severe somatic illness or neurological illness were excluded. Written informed consent was obtained from the subjects and the study was approved by the Ethics Review Committee on Human Research of the University of Tartu. Data were collected from 29.10.2001 to 27.03.2002.

An experienced clinician (SJ) assessed all the subjects to identify NIP cases in accordance with DSM-IV. The DSM-IV diagnostic criteria for other neuroleptic-induced movement disorders (NIMD) were also checked because of frequent comorbidity and common aetiology. Clinical NIP symptoms were assessed by SAS and the motor activity during rest was measured by actometry. Each item of the 10-item SAS is rated on a 5-point scale (0–4), and the mean score is obtained by adding the items and dividing by 10 [13]. Neuroleptic-induced akathisia and tardive dyskinesia were rated by Barnes Akathisia Rating Scale (BARS) [23] and Abnormal Involuntary Movement Scale (AIMS) [24].

The actometric recording was performed during sitting in a standardized clinical interview for 30 minutes, a method described previously as measuring "controlled rest activity" [19,21]. Controlled rest activity is a parameter of motor activity in a situation where sitting still is adequate and expected, but not instructed or required. The actometers (PAM3, Individual Monitoring Systems, Baltimore, USA) were attached to the ankles of the subjects to measure lower limb motor activity. Actometers are wireless, computerized movement detectors of match-box-size, which do not influence normal moving of the patient.

Cronbach's α was assessed to evaluate the internal consistency of the scale. The correlations between the lower limb activity (the mean of right and left ankle movement indices) and individual item scores and mean SAS scores were analysed. Differences between the NIP and non-NIP, as well as the NIMD and the non-NIMD groups in the SAS mean score and lower limb activity were analysed. The performance of SAS mean score and individual item scores in case identification was evaluated by receiver operating characteristics (ROC) analyses against DSM-IV NIP diagnosis. Validity coefficients (specificity, sensitivity, positive and negative predictive value [PPV and NPV, respectively]) for different mean SAS score thresholds were calculated. To explore the discriminatory power of each single SAS item we performed ROC analyses for each item separately. We also explored the effect on the validity coefficients of merging the six rigidity items of SAS into one single item, to de-emphasise the influence of rigidity on the mean SAS score. The Spearman test was used to correlation analysis and the Mann-Whitney 2-tailed U-test for the comparison between two groups because of the non-normal distribution of the data. The software used in analyses was SPSS 11.0. [25].

## Results

Of the 99 participants, 45 (45.5%) were male and 54 (54.5%) female. The mean age was 49.7 (SD 9.5) years. The mean continuous treatment in hospital or in nursing home was 13.6 (SD 9.0) years. Seventy-nine (79.8%) patients used conventional antipsychotics (70 on low-dose, and 9 on high-dose neuroleptics) and 20 (20.2%) used clozapine (one was receiving clozapine combined with sulpiride). Low-dose antipsychotics in this study were haloperidol, cyclopentixol, perphenazine and fluphenazine; high dose antipsychotics were chlorpromazine, thioridazine, levomepromazine, chlorprotixen and sulpiride. Sixteen (16.2%) patients were receiving combinations of typical antipsychotics (either predominantly low-dose [N = 10] or predominantly high-dose [N = 6] neuroleptic regimens), and 63 (63.6%) were receiving monotherapy (haloperidol: N = 29; zuclopenthixol: N = 28; perphenazine, chlorpromazine, or thioridazine: N = 6). No new atypical antipsychotics were used. The mean daily chlorpromazine equivalent conditions. The prevalence of any NIMD according to DSM-IV was 61.6% in the whole sample. Cronbach's α for SAS was 0.79. dose [26] was 328 (SD 221) mg. The prevalence of NIP according to DSM-IV criteria was 23.2%. Fourteen patients, all from non-NIP subgroup, used an anticholinergic drug (trihexyphenidyl). Only 10 of the 23 patients with NIP presented as pure NIP without comorbidity of other motor disorders. Among patients with NIP, 10 had comorbid akathisia and 6 tardive dyskinesia; three of them had all three The SAS mean score correlated significantly with age in our population (r = 0.203, p = 0.044).

### Convergence of SAS and actometry to DSM-IV NIP diagnosis

The SAS mean score for DSM-IV NIP patients (1.24, SD = 0.44) was significantly higher from that (0.56, SD = 0.33) of non-NIP patients (U = -6.90, p = 0.000). The mean scores of each single SAS item are presented in Table 1. The mean scores of "glabella tap" and "salivation" items for NIP patients were not significantly higher from that of non-NIP patients. The SAS mean score for NIMD patients was significantly higher from that of non-NIMD patients (U=-5.77, p = 0.000).

**Table 1:** Mean scores of Simpson-Angus Scale (SAS) items in Neuroleptic-Induced Parkinsonism (NIP) group and non-NIP group.

**SAS item**	**NIP-group**	**Non-NIP group**
Gait	1.04	0.38
Arm dropping	1.43	0.59
Shoulder shaking	1.09	0.33
Elbow rigidity	1.83	0.47
Wrist rigidity	0.91	0.16
Leg pendulousness	0.91	0.28
Head dropping	1.48	0.66
Glabella tap	1.09	0.86
Tremor	1.78	1.14
Salivation	0.83	0.71
		
Mean of SAS items	1.24	0.56

Actometric data was missing for one male patient due to non-co-operation. The median lower limb activity for NIP patients was not significantly higher than that of non-NIP patients (U = -0.46, p = 0.643). The median lower limb activity for NIMD patients was significantly higher from that of non-NIMD patients (U=-2.66, p = 0.008).

### Convergence of SAS to actometry

The SAS mean score did not correlate significantly with actometric lower limb activity either in the whole population (r = 0.04, p = 0.717), in the NIP group (r = -0.29, p = 0.192), or in the pure NIP subgroup (r = -0.21, p = 0.587). Even after a post-hoc analysis of co-variance in the whole population, where the effect of akathisia (BARS global score) and tardive dyskinesia (AIMS severity score) were controlled for, no significant correlation between SAS mean score and the lower limb activity could be found (r = 0.07, p = 0.494).

The tremor item of the SAS correlated significantly with the lower limb activity in the whole population (r = 0.25, p = 0.013) but not in the NIP population (r = 0.26, p = 0.248) or in the pure NIP subgroup (r = 0.51, p = 0.160). No correlation was evidenced between the hypokinesia item of the SAS and lower limb activity in the whole population (r = -0.07, p = 0.513) either in NIP population (r = -0.24, p = 0.290) or in pure NIP subgroup (r = -0.37, p = 0.797).

No correlation was evidenced between the mean of rigidity items of the SAS and lower limb activity in the whole population (r = -0.12, p = 0.256) either in NIP population (r = -0.37, p = 0.090) or in pure NIP subgroup (r = -0.30, p = 0.426).

### NIP case finding by SAS

**Figure 1:**
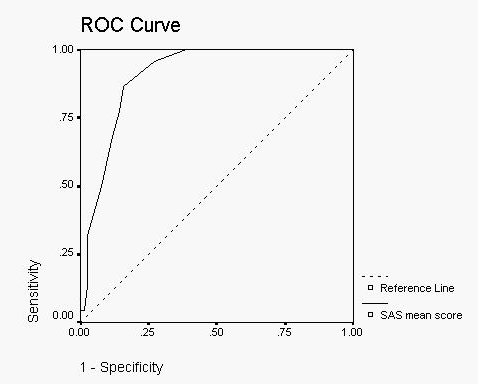
Receiver Operating Characteristic (ROC) curve for SAS mean score against DSM-IV defined Neuroleptic-Induced Parkinsonism (NIP).

ROC-curve for screening performance of SAS mean score is presented in Fig 1. Area under the ROC-curve (AUC) for SAS mean score was 0.92 (CI = 0.87–0.97). AUC of the ROC curve for SAS elbow rigidity item was 0.93 (CI = 0.86 – 1.0). AUC for the other items was less than 0.82. AUC in ROC analyses may range from of 0.5 (no case finding power) to 1.0 (optimal case finding performance). The validity coefficients of the SAS mean score are presented in Table 2.

**Table 2:** Validity coefficients of the Simpson-Angus Scale (SAS) mean score at different cutoff values. The optimal cut-off point range is presented in bold text.

SAS mean cut-off	0.25	0.35	0.45	0.55	**0.65**	**0.75**	**0.85**	0.95	1.05	1.15
Sensitivity	1.0	1.0	1.0	1.0	**1.0**	**0.96**	**0.87**	0.78	0.70	0.52
Specificity	0.17	0.36	0.45	0.49	**0.62**	**0.74**	**0.86**	0.86	0.89	0.93
Positive Predictive Value	0.27	0.32	0.35	0.37	**0.44**	**0.96**	**0.65**	0.64	0.67	0.71
Negative Predictive Value	1.0	1.0	1.0	1.0	**1.0**	**0.98**	**0.96**	0.93	0.91	0.87

ROC-curve for screening performance of SAS mean with single averaged rigidity item was clearly inferior to the original SAS mean curve with AUC of 0.80 (CI = 0.70–0.89).

The screening performances of the individual SAS items for NIP case finding are shown at Table 3.

**Table 3:** Area under the ROC curve of Simpson-Angus Scale (SAS) parameters against DSM-IV diagnosis of Neuroleptic-Induced Parkinsonism.

**SAS item**	**Area under the ROC curve**
Gait	0.71
Arm dropping	0.79
Shoulder shaking	0.81
Elbow rigidity	0.93
Wrist rigidity	0.75
Leg pendulousness	0.73
Head dropping	0.75
Glabella tap	0.57
Tremor	0.66
Salivation	0.53
	
Mean of rigidity items	0.92
Mean of mean rigidity items and other SAS items	0.80
Mean of SAS items	0.92

As SAS elbow rigidity item had case finding power similar to SAS mean score, we calculated optimal cut-off for this item. Cut-off threshold of 1.5, with sensitivity of 0.826 and specificity of 0.974, was superior to cut-off threshold of 0.5 with sensitivity of 0.957 and specificity 0.553.

## Discussion

Our study aimed to evaluate some of the characteristics of the SAS and its utility for identifying and measuring NIP in a naturalistic schizophrenia sample. The internal consistency of SAS was satisfactory, which suggests sufficient reliability for the scale. We compared the SAS with the DSM-IV to assess its discriminant validity and evaluate it in detecting NIP cases. The comparison with objective movement assessment aimed to estimate the concurrent validity of SAS in NIP severity measurement.

As expected, the SAS had discriminant validity for a clinical diagnosis of NIMD. SAS mean score discriminated NIMD patients well from those without NIMD, and more specifically, also NIP patients from other patients. Actometry discriminated NIMD patients from non-NIMD patients, but did not identify DSM-IV NIP patients.

According to ROC analysis the SAS had good case finding properties converging with the DSM IV NIP diagnosis. In our population the commonly used threshold 0.3 was inappropriate: according to our results the optimal cut-off point should be between 0.65 – 0.95 depending on the emphasis in the trade-off between sensitivity and specificity. We suggest that the new cut-off value for screening NIP could be 0.65, whereby specificity could be doubled without loosing any sensitivity. To be useful for diagnostic purposes a combination of high specificity and high positive predictive value (PPV) is reached at cut-off – 0.75 [27]. To answer to criticism about the overrepresentation of rigidity items, we averaged the six items into one item. This procedure worsened the NIP case detection capacity of the SAS.

Using the single elbow rigidity item for case detection had the same (or slightly better) case detection capacity as the SAS mean score. This finding supports the use of elbow rigidity testing when assessing parkinsonism in clinical settings, as cut-off value 0.5 has good sensitivity and specificity for DSM-IV NIP.

We found that SAS mean score did not correlate with actometric lower limb activity, and hypokinesia observed during gait item of SAS did not correlate with actometric motor activity during the 30-minute recording. There are a few explanations for that:

First, actometry measures only the productive motor dimension of the parkinsonian symptoms while SAS takes into account also rigidity, gait, salivation and glabella tap, with a clear emphasis on rigidity. Lack of correlation with actometric findings in NIP subgroup indicates that tremor may not be the core feature of NIP. This is also supported by the small AUC for the tremor item of SAS.

Secondly, we used lower-limb actometry while the clinical assessment by SAS and DSM-IV considered predominantly upper limbs. Parkinsonism may be more symptomatic in upper limbs, and the upper limb disturbances may have influenced our SAS and DSM IV assessments more than lower limb disturbances. Our findings indicate that lower limb actometry is not suitable for diagnosing NIP.

Thirdly, diurnal naturalistic actometry may have more power in detecting hypokinesia.

### Limitations

This study was limited to a few aspects of utility/validity of the SAS: internal consistency, convergence to DSM-IV NIP diagnosis and convergence to objectively measured motor activity. Many aspects of the scale's reliability (e.g. test-retest and inter-rater reliability) and validity (e.g. construct) were not evaluated.

DSM-IV was used as a standard in this study, but there is not much data available on the validity of NIP criteria of the DSM-IV. A better golden standard in this study would probably have been an expert-consensus diagnosis. Furthermore, as there was only one rater for the scales, a cross-scale contamination issue might have occurred.

It is known that with age the prevalence of spontaneous NIMD rises. Our material did not allow a thorough examination of the issue, but age correlated with SAS mean score in our sample.

The measurement of motor activity here was purely quantitative; we did not assess the patterns of the disordered movements.

## Conclusion

As a conclusion, SAS seems be a reliable and a valid instrument. It performs well and similarly to DSM-IV in NIP case detection. The optimal SAS mean score cut-off value in a naturalistic population of neuroleptic-treated schizophrenia patients is higher than the commonly used 0.3. We suggest that the new cut-off value for screening NIP could be 0.65, whereby specificity could be doubled without loosing sensitivity. Combining SAS rigidity items does not seem to improve the performance of the scale.

## Competing interests

The author(s) declare that they have no competing interests.

## Authors' contributions

SJ contributed to the study design, collected the data, contributed to the analyses and data interpretation, made the literature search and was responsible for manuscript preparation with MMH.

MMH contributed to study design, made most of the analyses and data interpretation, and was equally responsible for manuscript preparation with SJ.

KT contributed to study design, statistical analysis, data interpretation and manuscript preparation.

KW supervised the study design and contributed to statistical analysis, data interpretation and preparation of the manuscript.

## Pre-publication history

The pre-publication history for this paper can be accessed here:


